# Plant-Derived Bioactive Compounds in the Management of Neurodegenerative Disorders: Challenges, Future Directions and Molecular Mechanisms Involved in Neuroprotection

**DOI:** 10.3390/pharmaceutics15030749

**Published:** 2023-02-23

**Authors:** Shoaib Shoaib, Mohammad Azam Ansari, Adel Al Fatease, Awaji Y. Safhi, Umme Hani, Roshan Jahan, Mohammad N. Alomary, Mohd Nazam Ansari, Nabeel Ahmed, Shadma Wahab, Wasim Ahmad, Nabiha Yusuf, Najmul Islam

**Affiliations:** 1 Department of Biochemistry, Faculty of Medicine, Aligarh Muslim University, Aligarh 202001, Uttar Pradesh, India; 2 Department of Epidemic Disease Research, Institute for Research and Medical Consultations (IRMC), Imam Abdulrahman Bin Faisal University, Dammam 31441, Saudi Arabia; 3 Department of Pharmaceutics, College of Pharmacy, King Khalid University, Abha 62529, Saudi Arabia; 4 Department of Pharmaceutics, College of Pharmacy, Jazan University, Jazan 45142, Saudi Arabia; 5 Department of Botany, Faculty of Lifesciences, Aligarh Muslim University, Aligarh 202001, Uttar Pradesh, India; 6 Advanced Diagnostic and Therapeutic Institute, King Abdulaziz City for Science and Technology (KACST), Riyadh 11442, Saudi Arabia; 7 Department of Pharmacology and Toxicology, College of Pharmacy, Prince Sattam Bin Abdulaziz University, Alkharaj 11942, Saudi Arabia; 8 Department of Life Sciences, Shiv Nadar University, Greater Noida 201314, Uttar Pradesh, India; 9 Department of Pharmacognosy, College of Pharmacy, King Khalid University, Abha 61421, Saudi Arabia; 10 Department of Pharmacy, Mohammed Al-Mana College for Medical Sciences, Dammam 34222, Saudi Arabia; 11 Department of Dermatology, University of Alabama at Birmingham, Birmingham, AL 35294, USA

**Keywords:** neurodegenerative disorders, dementia, neuroinflammation, cholinesterase, amyloid β, antioxidant, plant formulations, phytocompounds

## Abstract

Neurodegenerative disorders encompass a wide range of pathological conditions caused by progressive damage to the neuronal cells and nervous-system connections, which primarily target neuronal dysfunction and result in problems with mobility, cognition, coordination, sensation, and strength. Molecular insights have revealed that stress-related biochemical alterations such as abnormal protein aggregation, extensive generation of reactive oxygen and nitrogen species, mitochondrial dysfunction, and neuroinflammation may lead to damage to neuronal cells. Currently, no neurodegenerative disease is curable, and the available standard therapies can only provide symptomatic treatment and delay the progression of the disease. Interestingly, plant-derived bioactive compounds have drawn considerable attention due to their well-established medicinal properties, including anti-apoptotic, antioxidant, anti-inflammatory, anticancer, and antimicrobial properties, as well as neuroprotective, hepatoprotective, cardioprotective, and other health benefits. Plant-derived bioactive compounds have received far more attention in recent decades than synthetic bioactive compounds in the treatment of many diseases, including neurodegeneration. By selecting suitable plant-derived bioactive compounds and/or plant formulations, we can fine tune the standard therapies because the therapeutic efficacy of the drugs is greatly enhanced by combinations. A plethora of in vitro and in vivo studies have demonstrated plant-derived bioactive compounds’ immense potential, as proven by their capacity to influence the expression and activity of numerous proteins implicated in oxidative stress, neuroinflammation, apoptosis, and aggregation. Thus, this review mostly focuses on the antioxidant, anti-inflammatory, anti-aggregation, anti-cholinesterase, and anti-apoptotic properties of several plant formulations and plant-derived bioactive compounds and their molecular mechanisms against neurodegenerative disorders.

## 1. Introduction

Globally, neurodegenerative disorders affect millions of people, imposing psychological and socio-economic burdens on the population, and epidemiological data indicate that nearly one in six of the world population suffer from neurodegenerative disorders. Dementia, in general, refers to a set of symptoms that impact a person’s memory, reasoning, and social abilities. Neurodegenerative diseases such as Alzheimer’s disease (AD), vascular dementia (VD), Lewy body dementia (LBD), frontotemporal dementia (FtD), and mixed dementia (MD) are known as progressive dementias, whereas Parkinson’s disease (PD), Huntington’s disease (HD), and traumatic brain injury (TBI) are linked to dementia. Dementia is prominently demarked by cognitive changes such as memory loss, disorientation, confusion, and difficulty with communication, coordination, organization, and motor functions, as well as psychological changes including depression, anxiety, paranoia, inappropriate behavior, and personality changes [1]. Current data on neurodegenerative diseases suggest that the world population may face greater risk of being affected by many neurodegenerative disorders in future. Late-onset-dementia prevalence estimates are rising exponentially with age, and epidemiological studies suggest that 45 million individuals have late-onset dementia, whereas young-onset-dementia prevalence estimates vary from 42.3 to 54 per 100,000 people [2]. Several factors contribute to dementia, including age and family history [3], alcohol and smoking [4], nutrition, physical activity and lifestyle [5], sleep disturbances [6], diabetes [7], cardiovascular health [8], and environmental risk factors [9]. Often, the majority of neurodegenerative diseases are strongly linked with aging, sharing aging-related biological hallmarks such as deregulated mitochondrial signaling, epigenetic modulations, genomic instability, cellular senescence, altered cell-to-cell communication, telomere shortening, and deregulated nutrient sensing [10]. Diagnosing dementia is a challenging task; biomarker-based diagnosis may provide accuracy but no single test is sufficient to diagnose dementia. Therefore, cognitive neuropsychological tests, brain (CT, MRI, and PET) scans, laboratory tests, and neurological and psychiatric evaluation may help in diagnosing dementias [11,12].

## 2. Methodology

In this review, we adhered to the preferred reporting items for systematic reviews and meta-analyses (PRISMA). PubMed, Web of Science, Science Direct, Scopus, and Google Scholar were used to search the literature for in vitro and in vivo investigations on the neuroprotective effects of plants and their phytocompounds. The ability of plant extracts to target acetylcholinesterase (AChE), butyrylcholinesterase (BChE), oxidative stress-, and neuroinflammation-marker proteins was screened. The major keywords used to search the literature in selected authentic databases were “plants”, “phytocompounds”, “phytochemicals”, “phytoconstituents”, “phytochemical profiling”, “bioactive compounds”, “dementia”, “dementia-related diseases”, “oxidative stress”, “inflammation”, “cholinesterase”, “antioxidant”, “neurodegenerative diseases”, “anti-cholinesterase activity”, “neuroprotection”, “amyloid β (Aβ)”, “AβO”, “Alzheimer’s disease”, “vascular dementia”, etc. This review article excluded book chapters, books, unpublished findings, and conference abstracts, and considered papers published in the English language only.

## 3. Plants and Their Bioactive Compounds in Averting the Pathogenesis of Neurodegenerative Disorders

Unfortunately, most dementias are not curable, but symptomatic treatment can be achieved by medications (cholinesterase inhibitors), occupational therapies, alternative medicines, and other methods. For example, donepezil, rivastigmine, and galantamine are prescribed to treat Alzheimer’s disease, as well as for vascular dementia, Lewy body dementia, and Parkinson’s disease [13]. Moreover, in recent decades, plants and their bioactive compounds have gained tremendous attention due to their excellent health benefits. A growing body of studies on plants and their bioactive compounds clearly suggests that they exert a number of biological actions, including neuroprotection [14], cardioprotection and hepatoprotection [15], anti-inflammation, anticancer and antioxidant action [16], antimicrobial action [17], and antidiabetic and other health-beneficial actions [18]. The primary focus of this review is on the possible functions of plants and their phytocompounds in the management of neurodegenerative disorders. Table 1 and Table 2 summarize the effects of plants and bioactive compounds, respectively.

### 3.1. Exploring Antioxidant, Anti-Acetylcholinesterase, and Anti-Butyrylcholinesterase Activity of Plants and Their Compounds in Neurodegenerative Disorders

Blueberries are members of the *Viccinium* genus, and many of the species are high in polyphenols, which have been demonstrated to have powerful antioxidant properties. Blueberry extracts protect microglia cells, reduce neuroinflammation, and slow the progression of dementia [19]. Phenolic compounds from flower extracts of Acacia dealbata showed significant biological activities, including antioxidant and inhibitory effects against acetylcholinesterase (AChE) in dementia [20]. Eighteen compounds were identified in the leaf extracts of *Sophora secundiflora* and *Sophora tomentosa* using LC-ESI-MS/MS analysis. In vivo investigations revealed promising neuroprotective benefits by lowering AChE, nor-adrenaline, and dopamine levels and increasing glutathione and acetylcholine levels [21]. Eugenol, β-elemene, eugenyl acetate, and methyl eugenol were found in essential-oil fractions of *Piper divaricatum* and were found to have strong inhibitory effects against AChE [22]. Phytochemical analysis of *Lavandula stoechas* (L.) ensured the presence of phenolics, flavonoids, and tannins, and methanolic extract of the plant showed potent free-radical-scavenging activity with an IC_50_ value of 76.73 μg/mL, declined brain AChE and malondialdehyde levels, and improved glutathione (GSH), catalase, and superoxide dismutase (SOD) levels, suggesting attenuation of dementia in scopolamine-induced memory-deficit mice [23]. Similarly, the phenolic and flavonoid contents of *Elatostema papillosum* showed significant antioxidant and inhibitory activity against AChE and butyrylcholinesterase (BChE) in Alzheimer’s disease, with IC_50_ values of 165.40 4.01 and 213.81 3.57 μg/mL, respectively [24]. Flavonoids, phenolics, and tannins from water extract of *Evolvulus alsinoides* (Linn.) showed antioxidant activity and AChE inhibition, with IC_50_ values of 52.43 ± 0.2 μg/mL and 4.46 ± 0.03 μg/mL, respectively [25].

*Psychotria calocarpa* leaf extract was recently studied for its cytotoxic, neuropharmacological, thrombolytic, antidiarrheal, and anxiolytic properties. Furthermore, extract from the leaves of *Psychotria calocarpa* demonstrated antioxidant activity in an in vitro and in vivo experiment, with an IC_50_ value of 461.05 μg/mL determined by a DPPH–free-radical-scavenging test and exerted a dose-dependent reduction in depressant behavior, which was attributed to the phenolic and flavonoid contents of the plant leaves [26]. In experimental animals, chronic arsenic exposure is typically associated with memory impairment, depression, and hepatotoxicity. As a result, various plants have been examined for their protective properties. One such in vivo study showed that juice from the leaves of *Morus alba* had significant protective effects against arsenic-induced neurotoxicity by reducing AChE and BChE level and improved hepatic functioning by restoring normal levels of GSH, SOD, alanine transferase (ALT), aspartate transferase (AST), and alkaline phosphatase (ALP) in Swiss albino mice [27]. The high-performance liquid chromatography (HPLC)–photodiode array (PDA) method revealed the presence of gallic acid, catechin, ellagic acid, and quercitrin in *Bauhinia coccinea*, and its ethanolic extract demonstrated neuroprotection through antioxidant activities, as evidenced by the significant inhibition of amyloid-β (Aβ) aggregation and AChE and suppression of hydrogen peroxide (H_2_O_2_)-induced cell death in HT22 neuronal cells in AD [28]. Among many extracts tested for anti-AD activities, chloroform extract from *Enhydra fluctuans* was reported to possess the best inhibitory activities, with IC50 values of 83.90 μg/mL and 48.14 μg/mL against AChE and BchE, respectively, and free-radical-scavenging activity was also observed at an IC50 value of 113.27 μg/mL by DPPH radical-scavenging assay, owing to the presence of 19.16 mg gallic acid equivalent/g extract of phenolics and 41.84 mg catechin equivalent/g extract of flavonoids in the extract [29].

Analysis revealed three triterpenoids and three flavonoids in *Dillenia suffruticosa* leaves, and among those six phytoconstituents, betulinic acid, koetjapic acid, and kaempferol had the highest inhibition activity against AChE and BChE, whereas the others showed relatively low potency in *Caenorhabditis elegans* [30]. In addition to AChE inhibition, antioxidant potential of *Rosmarinus officinalis* was reported withIC_50_ values of 272 µg/mL, 387 µg/mL, and 534 µg/mL for ethyl-acetate, ethanol, and aqueous extracts, respectively [31]. A recent in vivo study on *Euterpe oleracea* found that its extract reduced vascular dementia-induced problems as well as hippocampus mortality in CA1 and CA3 areas, perhaps owing to regulation of nuclear factor erythroid 2-related factor 2 (Nrf-2) and Beclin-1 activities [32]. In a recent study, TLC and Ellman’s colorimetric assay were used to test the effects of 90 extracts from 30 medicinal plants on AChE activity in AD at different concentrations (62.5, 125, and 250 µg/mL). Out of the 90 extracts, only 21 demonstrated greater anti-AChE activity (75–100% inhibition), whereas oregano extract showed the highest antioxidant activity as measured by DPPH scavenging assay. TLC, HPLC-DAD, and LC-MS identified 23 compounds, including the most abundant as flavonoids and dihydroxycinnamic acids [33]. Amyloid beta (Aβ1–42) elevated the hippocampal levels of TNF-α, MDA, and IL1β; augmented microgliosis; and diminished antioxidant status. For this reason, crude flavonoids and saponins from *Bacopa floribunda* were investigated and the results showed suppression of oxidative stress, neuroinflammation, and microgliosis [34]. Notably, out of 200 African plants tested, extracts from *Ammocharis coranica*, *Lannea schweinfurthii*, *Scadoxus puniceus* (L.), and *Xysmalobium undulatum* (L.) inhibited AChE with IC_50_ values less than 1 μg/ml in AD [35]. In rats with AF64A-induced memory deficits, a blend of *Cyperus rotundus* and *Zingiber officinale* extract improved memory and neuronal density while decreasing oxidative stress and AChE levels via up-regulation of pERK1/2, implying combined neuroprotective effects of *Cyperus rotundus* and *Zingiber officinale* in dementia [36].

GC-MS analysis of *Typha domingensis* disseminated that methanolic extract’s total phenolic and flavonoid content was 95.72 ± 5.76 mg GAE/g and 131.66 ± 7.92 mg QE/g, respectively, and it exhibited inhibitory activity against AChE and BChE and antioxidant activity measured by ferric-reducing antioxidant power (FRAP) and DPPH scavenging assays [37]. Notably, two acetylcholinesterase (AChE) inhibitors were identified in the peel extract of *Annona cherimola* through HPTLC–bioassay–mass spectrometry (MS) and HPLC-DAD-MS/MS [38]. In an in vitro experiment, betulinic acid, ursolic acid, jacoumaric acid, corosolic acid, and daucosterol were recovered from *Syzygium antisepticum* ethyl-acetate extract and gallic acid, myricitrin, and quercitrin from methanolic extract. The researchers showed that methanolic extract exhibited strong antioxidant and anti-AChE activities. Notably, methanolic extract exerted antioxidant activity in HEK-293 cells by suppressing hydrogen peroxide-induced reactive oxygen species and elevated the levels of antioxidant enzymes such as catalase, GSH, glutathione peroxidase-1 (GPx-1), and glutathione reductase [39]. *Dracaena reflexa* phytochemical screening resulted in the detection of many bioactive phytoconstituents by GC-MS, and the total phenolic and flavonoid contents in the extracts were 92.72 ± 0.79 mg GAE/g extract and 110 ± 0.83 mg QE/g extract, respectively. With respect to other fractions, the n-butanol fractions demonstrated considerable inhibitory activity against tyrosinase and AChE, as well as antioxidant activities in the DPPH free-radical-scavenging and FRAP assays [40]. Several phytocannabinoids, flavonoids, and terpenes of *Cannabis sativa* (L.) displayed neuroprotective effects in neurodegenerative conditions and exemplified antioxidant and anti-amyloid β aggregation actions against pathological toxic hallmarks in AD [41].

Liquid chromatography–mass spectrometry (LC-MS) and gas chromatography–mass spectrometry (GC-MS) of leaf extract from *Solanum macrocarpon* (L.) showed 78 and 60phytoconstituents, respectively, and among different fractions, the ethyl-acetate fraction displayed potent antioxidants and concentration-dependent AChE inhibition in AD [42]. A total of 49 bioactive phytocompounds in dried and ripe fruits of *Alpinia oxyphylla* and ripe fruits of *Alpinia oxyphylla* Miquel were identified using ultra-high-performance liquid chromatography with triple quadrupole mass spectrometry, with 19of them exhibiting anti-BChE activity in AD [43]. The phytoconstituents in *Bruguiera gymnorhiza* (L.) decoctions were identified using ultra-high-performance liquid chromatography/electrospray ionization tandem mass spectrometry (UHPLC-ESI-MS/MS), and then further inquests revealed their inhibitory activity on tyrosinase, AChE, and BChE, as well as antioxidant activity measured by DPPH and FRAP assays. Notably, phenol-, flavanol-, tannin-, and triterpenoid-rich decoction revealed high antioxidant capacities (547.75 ± 10.99 mg TE/g), but inhibitory values for AChE, BchE, and tyrosinase were 3.75 ± 0.03, 2.19 ± 0.13 mg GALAE/g, and 147.01 ± 0.78 mg KAE/g, respectively [44]. GC-MS was used to detect multiple bioactive constituents in the leaves and essential oils of *Artemisia scoparia* and *Artemisia absinthium*. Only nine phytochemicals were found to have substantial antioxidant activity with an IC_50_ value of 285 ± 0.82 µg/mL, whereas oils showed some good inhibitory action against AChE and BChE, with IC_50_ values of 30 ± 0.04 µg/mL and 34 ± 0.07 µg/mL, respectively [45]. Furthermore, the phytochemical analysis, antioxidants, and cholinesterase inhibitory activity of *Mentha pulegium* (L.), a plant growing in Bosnia and Herzegovina, were studied. The findings revealed that the plant extract contains ellagic acid, eriodictyol, naringenin, and chlorogenic acid, as well as significant antioxidant activity and the ability to neutralize lipid peroxidation and protein oxidation, but has little or no inhibitory influence against AChE and BChE [46]. 

Phytochemical analysis confirmed the presence of β-sitosterol, 3-O-β-acetyloleanolic acid, betulinic acid, and oleanolic acid in dichloromethane and ethyl-acetate fractions of *Lawsonia inermis* (L.) seeds, which exhibited selective inhibitory action against BChE, with IC_50_ values of 113.47 and 124.90 μg/mL, respectively, whereas ethyl-acetate and methanolic extracts displayed excellent antioxidant capacity, with IC_50_ values of 3.08 μg/mL and 3.64 μg/mL, respectively [47]. *Ferula ammoniacum*, an endemic medicinal plant, has been reported to contain epigallocatechin gallate (EGCG), ellagic acid, quercetin, chlorogenic acid, phloroglucinol, mandelic acid, hydroxy benzoic acid, malic acid, rutin, and pyrogallol in different fractions. Ethyl acetate and chloroform extracts showed potent inhibitory activity against cholinesterases (AChE and BchE), with IC_50_ values of 40 and 43 µg/mL, and 41 and 42 µg/mL, respectively, whereas DPPH assay showed that the ethyl-acetate fraction exerted considerable antioxidant activity, with an IC_50_ value of 100 µg/mL [48]. *Ginkgo biloba* is a plant with medicinal properties that has been reported to improve dementia, eye problems, anxiety, and peripheral artery disease. LC/MS analysis of *Ginkgo biloba* identified many phenylpropanoid glycosides out of isolated compounds, and a few showed greater radical scavenging activity with anIC_50_ value of 5.23 μM [49]. Aqueous extract of fruits from the *Morus* plant species was demonstrated to possess phenolics including cyanidin, kuromanin, and keracyanin, and the extract showed anti-AD properties by exhibiting antioxidant activity in vitro [50].

LC–MS/MS analysis of *Salvia eriophora* leaves confirmed salvigenin and fumaric acid in the ethanol extract, and fumaric acid, caffeic acid, and epicatechin in the water extract of the plant. Furthermore, the extracts showed inhibitory properties against AChE, BChE, α-amylase, and α-glycosidase, as well as antioxidant activity [51]. HPLC-DAD-ESI/HRMS displayed the presence of 73 compounds in 60ethanolic extracts of *Origanum majorana*, *Origanum onites*, *Origanum syriacum*, *Origanum hirtum*, and *Origanum viride*. The results of the investigated extracts showed good anti-AChE and anti-BChE activity, whereas among the detected phytocompounds, aromadendrin exhibited potent reducing power and inhibitory activities against AChE and BChE [52]. Of note, *Folium perseae* (avocado) leaves were also examined for their neuroprotective properties and the investigations showed their antioxidant and anticholinergic effects through the suppression of AChE and BChE [53]. Ginkgolide B, a common phytoconstituent of *Ginkgo biloba* and *Machilus wangchiana*, was studied for its neuroprotective effects. Recently, one such study highlighted that ginkgolide-B exposure to human neuroblastoma SH-SY5Y cells protected against Aβ_1–42_-induced oxidative damage by reducing ROS generation, protein-carbonyl content, and lipid peroxidation, whereas ginkgolide B reversed antioxidant status to normal by enhancing SOD and GSH levels [54].

### 3.2. Exploring Anti-Apoptotic and Anti-Inflammatory Activities of Plants and Their Compounds in Neurodegenerative Disorders

An in vivo study on prophylactic treatment with extract of black pepper β-caryophyllene (ViphyllinTM) showed improved cognitive functions by suppressing Scopolamine-induced upregulation of inducible nitric-oxide synthase (iNOS), bax, caspases, p-JNK, and p-38-MAPK, whereas it augmented expression of bcl-2 and TrkB in Scopolamine-induced dementia-model mice [55]. Sinensetin, an active phytoconstituent of many citrus plants, displayed anti-inflammatory, antioxidant, and antiapoptotic activities against amyloid beta (Aβ_25–35_)-induced neurotoxicity. Notably, pretreatment with sinensetin for an hour, followed by co-treatment with Aβ and sinensetin, attenuated oxidative stress, inflammation, and apoptosis, and improved cell survival through targeting the expression of nuclear factor-kappaB (NF-κB) and Toll-like receptor-4 (TLR4) proteins in SH-SY5Y cells [56]. It is a well-established fact that hydrogen peroxide significantly elevates ROS generation within cells, thereby promoting apoptosis induction. On the contrary, stigmasterol has been demonstrated to prevent ROS-dependent apoptosis induction through upregulating bcl-2, forkhead box O (FoxO) 3a, and catalase in neurons [57]. *Petroselinum crispum* extract increased bcl-2 and M1 receptor expression while also lowering caspase-3/-9, bax, AChE activity, and malondialdehyde (MDA) levels in the hippocampus and frontal cortex, indicating neuroprotective effects of plant extract against a scopolamine-induced Alzheimer’s -disease (AD) rat model [58]. *Cyprus rotundus* is a medicinal herb with several applications in the food and confectionary industries, and recently *Cyprus rotundus* rhizome extract (CRE) was investigated for its neuroprotective properties. Pretreatment with CRE significantly attenuated hydrogen peroxide-induced damage to SH-SY5Y cells through its antioxidant and anti-apoptotic mechanisms [59]. *Guazuma ulmifolia*, *Limonium brasiliense*, *Paullinia cupana*, *Poincianella pluviosa*, *Stryphnodendron adstringens*, and *Trichilia catigua* ethyl-acetate fractions were tested for antioxidant and anti-AChE activity against Aβ_25–35_-induced toxicity in SH-SY5Y cells. The ethyl-acetate fraction of *Stryphnodendron adstringens* substantially inhibited mitochondrial depolarization, superoxide generation, and Aβ_25–35_-induced lipid peroxidation [60]. Extract from the leaves of *Ocimum basilicum* (L.) containing 5,7-dihydroxy-3′,4′,5′-trimethoxyflavone and 3-hydroxy-3′,4′,5′-trimethoxyflavone mitigated cognitive impairment by reducing the concentrations of caspase-3, interleukine-6, interleukine-1β, TNF-α, and AChE activity in scopolamine-induced mice, and elevated levels of glutathione and IL-10 were also recorded in the hippocampal region of the brain [61].

**Figure 1:**
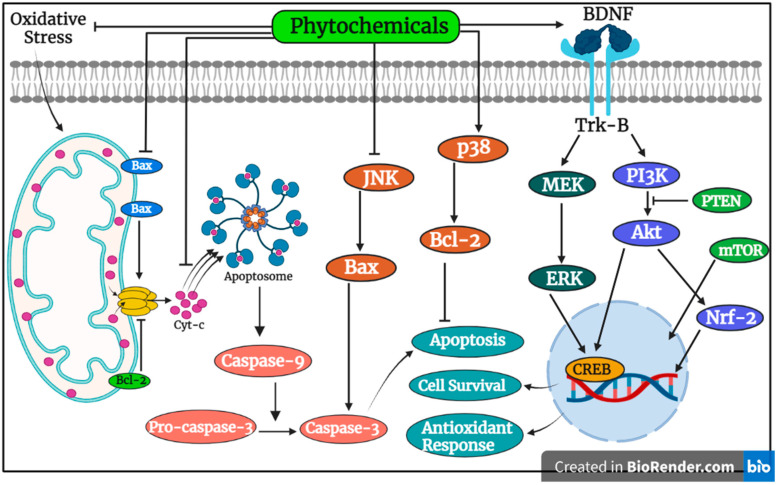
Apoptosis is triggered through the activation of intrinsic as well as extrinsic pathways involving modulation of several pro- and anti-apoptotic proteins. Phytochemicals can up-regulate anti-apoptotic proteins such as Bcl-2 while down-regulating expression of pro-apoptotic proteins, including bax, cytochrome-c, caspase-9, and caspase-3, in neurodegenerative diseases. Phytochemicals also enhance the expression of pro-survival-related proteins as well as Nrf-2, which leads to antioxidant response.

Shikonin administration by gavage to the rat model of vascular dementia at a dose of 10 mg/kg/day resulted in improved morphological changes in the CA1 region of the hippocampal neurons by down-regulating the expression of p-Akt, p-PTEN, p-CREB, and BDNF. Subsequently, shikonin was also reported to attenuate apoptosis induction by alleviated expression of bcl-2 and reduced bax expression in a dose-dependent manner [62]. Ursolic acid has long been studied for its neuroprotective properties against a myriad of neurodegenerative illnesses, such as chemical-induced cognitive loss and neurotoxicity, traumatic brain damage, cerebral ischemia and reperfusion injury, and subarachnoid hemorrhage. Mechanistic insights indicate that ursolic acid elevated the expression of FoxO1, Akt, Nrf2, SOD, GPx, CAT, and GR while down-regulating expression of TLR4, ICAM-1, IL-1β, TNF-α, NF-κB P65, IL-6, iNOS, and MMP-9, which augmented antioxidation, apoptosis inhibition, and inflammation attenuation. In a recent study, rosmarinic acid and ursolic acid were demonstrated to exert protective effects by improving spatial and recognition memory as well as anxiety in Aβ_1–42_-induced BALB/c mice [63]. Gastrodin, a bioactive phytochemical from *Gastrodia elata*, has been shown to effectively treat epilepsy, dizziness, dementia, and ischemic stroke. In an in vivo study, oral intake of gastrodin reduced apoptosis by modifying the expression of MAPK, bax, and bcl-2, and reduced autophagy by influencing the expression of beclin-1, p62, and LC3-II. Gastrodin reduced beta-amyloid (Aβ_1–40/42_) accumulation and alleviated cognitive impairment caused by bilateral common carotid-artery occlusion (BCCAO), as well as hippocampal CA1 and CA3 pyramidal neuronal damage in vascular dementia [64]. Another study indicated that gastrodin treatment suppressed ferroptosis induction and improved learning ability and memory impairment in rats with vascular dementia and the basis for the neuroprotective effects was suggested as gastrodin-decreased levels of Fe^2+^ and MDA and elevated GSH content by upregulation of Nrf2 and GPx4 and down-regulated Cox2 and kelch-like ECH-associated protein (Keap1) [65]. Quercetin induced reversal of memory impairment by alleviating the activity of IL-6 and TNF-α, suppressing Scopolamine-induced cell death, and degeneration in hippocampal sub-regions and prefrontal cortex in the brain of AD-model mice [66]. Figure 1 shows the role of phytochemicals in the suppression of apoptosis induction.

Ampelopsin A, a common bioactive component of Vitis vinifera, was shown to restore Scopolamine-induced long-term potentiation impairment in hippocampal CA1 and CA3 synapses in C57BL/6 mice, which was achieved by modulating BDNF/CREB signaling pathways; thereby, ampelopsin A exhibited neuroprotective and neurocognitive effects [67]. *Centella asiatica* is the main source of asiaticoside, which showed significant improvement in altered behavior and impairment in vascular dementia. Additionally, asiaticoside was found to be associated with the mitigation of hippocampal-tissue damage and formation of autophagosomes through increasing the expression of beclin-1 and microtubule-associated protein light chain 3 (LC3)-II and decreased phosphorylation of mTOR in rats [68]. Rosiridin, a natural monoterpene produced in *Rhodiola rosea*, has been demonstrated to affect oxidative stress and neuroinflammatory markers in rats by exhibiting anti-AChE activity and restoring normal levels of GSH, MDA, SOD, IL-6, IL-1, TNF-, caspase-3/-9, and IFN-γ [69]. A recent study found that methanolic, aqueous, and chloroform extracts of *Glaucium corniculatum* have neuroprotective properties against hydrogen peroxide-induced neuronal injury in PC12 cells. Several alkaloids were reported to be in all three solvents, and the antioxidant and anti-apoptotic effects of chloroform-alkaloid extract were linked to the inhibition of intracellular ROS formation and the alleviation of expression of bax and caspase-3/-9, respectively [70]. Another study examined the possible neuroprotective effects of rehmannioside A (isolated from *Rehmanniae* Radix) against vascular dementia. The mechanistic insights revealed that reduced oxidative stress, inflammation, and apoptosis induction were due to rehmannioside A-mediated activation of Nrf2 and attenuated expression of caspase-3 and NF-κB in a mouse model of vascular dementia [71]. In a study using a mouse model of vascular dementia, rats were administered 50 and 100 mg/kg *Panax ginseng* extract for eight weeks, leading to significant improvement in behavioral function and augmented neuronal density, which possibly occurred in response to elevated expression of VEGF and (FGF) [72]. 

*Dracocephalum moldavica* (L.) is a rich source of tilianin, and investigations on tilianin have shown its anti-apoptotic, anti-neurodegenerative, and antioxidant benefits, as well as improved cognitive impairment, which were attributed to restoration of ERK1/2 and CREB signaling and impeding JNK-, MAPK-, p38-, and NF-κB-related inflammatory responses in rats with vascular dementia [73]. Kaempferol, which was isolated from the leaves of *Mespilus germanica* (L.), has been studied for its potential role in Alzheimer’s disease, together with its impact on oxidative stress, neuroinflammation, apoptosis, lipid peroxidation, and cognitive impairment in an ovariectomized rat model of sporadic Alzheimer’s disease. They observed that kaempferol increased spatial learning and memory, increased antioxidant status by increasing GSH and SOD levels, and lowered tumor necrosis factor-α activity and malondialdehyde levels in the rats’ brains [74]. Marinoid J, a phenylethanoid, is a key phytoconstituent of *Avicennia marina*, and showed neuroprotective effects by reducing MDA levels and NO activity while augmenting glutathione-peroxidase content in the tissues of the hippocampal region and ameliorating cognitive impairment in vascular dementia [75]. Morin, a polyphenolic compound, is isolated from the members of the Moraceae family and has long been widely exploited for its range of biological actions, including neuroprotection, antioxidation, anti-aggregation, and anti-inflammation abilities. One such study has shown that morin and MK-801 combination treatments abated expression of dementia-related proteins, including Aβ_42_, APO-E, tau, and β-catenin phosphorylation, and exerted anti-inflammatory actions by altering the activity of IL-6, TNF-α, caspase-3, and NF-κB. Furthermore, it was demonstrated that morin and MK-801 combined post-treatment resulted in behavioral improvements; altered apoptosis, autophagy, and inflammatory responses; and conferred neuroprotective benefits in a rat model of mild repeated traumatic brain injury [76]. A recent study delved into whether *Perilla frutescens* leaf extract may improve vascular dementia. In all of these experiments, extracts at concentrations of 30, 60, and 90 mg/kg were orally administered perioperatively for 23 days, resulting in reversal of IL-6, TNF-α, and NO, as well as NF-κB, MAPK, and iNOS activities after a 12 h pretreatment with extract in lipopolysaccharide-induced neuroinflammation in rats [77]. Another recent finding on the neuroprotective effects of *Salvia macilenta* on Aβ-injected male albino Wistar rats found that a 50 mg/kg/day oral dosage of *Salvia macilenta* for 10 days reduced apoptosis, raised GSH and Nrf2 levels, and decreased TNF-α and IL-6 levels in the hippocampus and prefrontal cortex [78]. The studies explored whether glycyrrhizic acid protects against cognitive impairment in chronic cerebral hypoperfusion, attempting to find whether it would have potent enzymatic and non-enzymatic antioxidant activity by fixing intracellular ROS generation, as well as inhibiting cytochrome-c release to prevent apoptosis induction. Finally, it was shown that glycyrrhizic-acid treatment led to improvement of pyramidal neurons, myelin, and dendritic-spine density in a mouse model of vascular dementia [79].

### 3.3. Exploring Anti-Amyloid β (Aβ) Activity of Plants and Their Bioactive Compounds in Neurodegenerative Disorders

**Figure 2:**
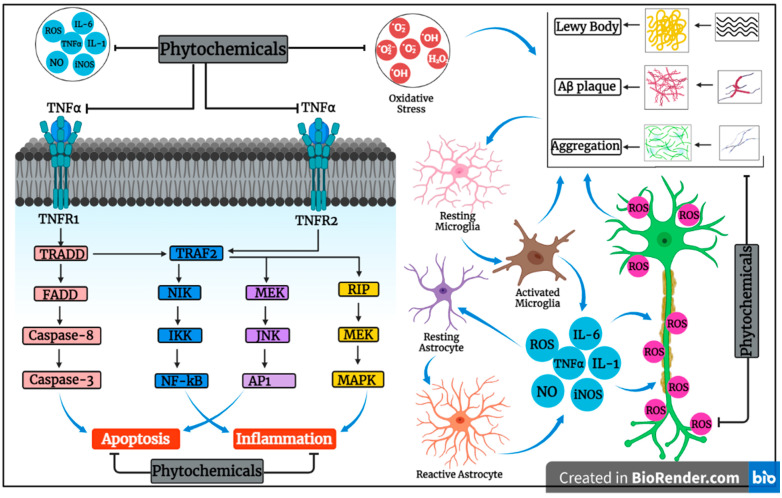
Oxidative stress due to the production of reactive oxygen species may lead to neurodegeneration. Activated microglia and reactive astrocyte generate ROS, pro-inflammatory cytokines, and inducible nitric-oxide synthase (iNOS), which may cause further damage. Binding of TNF-α to its receptor can lead to the activation of many signaling pathways, which may cause apoptosis induction and inflammation. On the other hand, phytochemicals have great ability to target apoptosis, inflammation, and oxidative stress, as depicted in the figure.

Alzheimer’s disease is mainly characterized by the deposition of oligomeric assemblies of amyloid β-protein (AβO), which leads to neurotoxicity and aggravates tau abnormalities, oxidative stress, and synaptic disturbances. For these reasons, numerous phytoconstituents have been investigated for their neuroprotective effects, as depicted in Figure 2. Tyrosol and hydroxytyrosol from olive oils inhibited AβO-dependent caspase-3 activation, whereas tyrosol did not affect AβO aggregation [80]. Interestingly, another study suggested that oral administration of hydroxytyrosol led to attenuation of spatio-cognitive deficits and AβO-induced deregulation of JAK2/STAT3, PI3K/Akt, ERK-MAPK, and JNK-p38 signaling were reversed by the compound [81]. Several researchers showed that honokiol exhibited neuroprotective effects, as honokiol administered intraperitoneally up to 14 days resulted in improvement in spatial-learning impairments in a dose-dependent manner and suppressed apoptosis and neuronal damage in the CA1 region of the hippocampus, and another study also presented honokiol as an anti-apoptotic agent because it suppressed AβO-induced apoptosis through inhibition of ROS production and attenuation of NF-κB signaling pathway in AβO-treated neurons [82]. Oral administration of ferrulic acid was demonstrated to reduce AβO deposition in the cerebral region, and ferrulic acid diminished cognitive impairment in a mouse model, whereas antioxidant effects of ferrulic acid against AβO were attributed to the activation of Nrf2 via ERK1/2 pathway [83,84]. Other investigators have reported ferrulic acid to be neuroprotective against Aβ_42_-induced neurotoxicity and oxidative stress in rat primary cortical neurons, whereas another found ferulic acid to be anti-apoptotic against AβO-induced cell death in neuroblastoma cells [85,86]. In a rat model of permanent bilateral common carotid-artery-occlusion (2VO)-induced vascular dementia, gastrodin was indicated to ameliorate memory impairment and executive dysfunction. The study also showed that gastrodin, at a dosage of 90 mg/kg/day, could decrease the formation of Aβ_1–40_ and Aβ_1–42_ plaques in the plasma and hippocampus of 2-VO rats by reducing tau and Aβ phosphorylation [87]. Naringenin, a common flavanone, is widely distributed in many citrus fruits, showing neuroprotective effects against Aβ_1–42_ evoked neurotoxicity by restoring AMPK levels and abating Aβ concentration in neuronal cells of mice, and displayed autophagy-inductive ability by alleviating beclin-1 ATG5 and ATG7 in Neuro2a cells and primary mouse neurons [88].

A novel sesquiterpenoid, Pocahemiketone A from *Pogostemon cablin*, was shown to alleviate NLR -family pyrin domain-containing 3 (NLRP3) inflammasome-dependent pyroptosis and oxidative stress, suggesting neuroprotective effects against Aβ_25–35_-mediated damage in SH-SY5Y cells [89]. Myricetin and dihydromyricetin, flavonoids present in many fruits and vegetables, have been shown to have an array of biological effects, such as antioxidant, anti-neuroinflammation, and inhibitory efficacy against Aβ oligomers in AD. Myricetin treatment resulted in the suppression of Fe^2+^-induced cell death in SH-SY5Y cells and showed anti-AChE activity and reversed Scopolamine-induced cognitive deficits in a mouse model [90]. Sulforaphane, an isothiocyanate found in cruciferous vegetables, was studied for its neuroprotective properties in a mouse model with AD lesions caused by a combination of D-galactose and aluminum. Further investigations of sulforaphane by these researchers showed amelioration of spatial cognitive impairment and attenuation of Aβ plaques in the cerebral cortex and hippocampal regions of sulforaphane-administered AD-lesion mice, whereas the mechanistic insights revealed that sulforaphane elevated glutathione peroxidase RNA expression and prevented Aβ deposition and peroxidation in mice with Alzheimer-like lesions [91]. Ethanolic extract of the leaves of *Elaeagnus glabra* f. *oxyphylla* decreased the formation of Aβ plaques at an IC_50_ value of 32.01 µg/mL and attenuated oxidative stress at an IC_50_ value of 12.32 µg/mL in AD. They also identified 16phytocompounds, with procyanidin B3, procyanidin B4, and helichrysoside exerting substantial anti-Aβ aggregation effects at IC_50_ values of 14.59, 32.64, and 44.45 μM, respectively [92]. Recently, rosmarinic acid in *Salvia fruticosa* was shown to have inhibitory action against Aβ_1–42_-induced cytotoxicity on SH-SY5Y cells by down-regulating glycogen synthase kinase (GSK) 3β and β-secretase activation at IC_50_ values of 6.52 ± 1.14 and 86 ± 2.9 µg/mL, respectively [93]. Another study showed that oral administration of extract of qingyangshen (Chinese herbal medicine) ameliorated learning ability and spatial memory. The researchers suggested that neuroprotective effects occurred in response to the inhibition of astrocytosis, microgliosis, and aggregation of Aβ and tau proteins while augmenting poly-ADP ribose polymerase (PARP) expression in the brains of transgenic mice [94].

Several triterpene saponins were isolated from the methanolic extract of *Stenocereus pruinosus*, and thioflavin-T assay revealed the significant inhibitory power of triterpenes against Aβ aggregation [95]. Various flavonoids, isoflavonoids, and coumestan are widely distributed in *Pueraria lobata*. In a recent study, coumestrol, an active phytoconstituent of *Pueraria lobata* leaves, displayed anti-Aβ aggregation and selective inhibition of monoamine-oxidase activation at an IC_50_ 1.99 ± 0.68 µM, suggesting neuroprotective effects of the active compound against AD [96]. Ginsenoside F1, an active component in *Panax ginseng*, was studied for its neuroprotective properties, together with its ability to reduce amyloid beta aggregation in Alzheimer’s disease. Ginsenoside F1 at 2.5 µM inhibited Aβ aggregation in SH-SY5Y neuronal cells in an in vitro study. Besides that, after two hours of post-treatment, ginsenoside F1 was found to be capable of crossing the blood-–brain barrier, with elevated levels of insulin-degrading enzyme and neprilysin proteins after eight weeks of administration of 10 mg/kg/d ginsenoside F1 and reduced Aβ plaques in mice models of Alzheimer’s disease (AD) [97]. *Pandanus amaryllifolius* has wide distribution in Southeast Asia and is exploited for its health-benefit effects, including vitamins, antioxidants, and anti-diabetic and anticancer properties. Notably, crude-alcoholic extract and crude-base extract of *Pandanus amaryllifolius* in doses of 50 μg/mL led to obstructed Aβ oligomerization and deposition, increased cell survival, suppression of ROS generation, and restored mitochondrial functions at different doses in SY-SY5Y cells [98].

*Carthamus tinctorius* (L.) and *Taraxacum coreanum* are common traditional remedies in Asian nations, and their antioxidant and anti-inflammatory properties have been studied. *Carthamus tinctorius* (L.) seeds and *Taraxacum coreanum* were shown in a recent study to improve cognitive dysfunction, learning, and memory abilities synergistically when compared to a single therapy. Interestingly, both plants suppressed the production of amyloidogenesis-associated proteins such as β-secretase and γ-secretase in Aβ_25–35_-infused rats [99]. Interestingly, terpenes such as β-caryophyllene and α-bisabolol at a dose up to 100 µM showed significant antioxidant activity and enhanced cell survival against Aβ exposure, and inhibited Aβ_1–42_ fibril formation and deposition [100]. Cirsium japonicum var. maackii is a medicinal herbal plant with antibacterial and anti-inflammatory properties. In a recent study, an ethyl-acetate fraction from the plant demonstrated neuroprotective effects, evidenced by improved spatial memory and object recognition in comparison to the untreated group. They also demonstrated a dose-dependent reduction in lipid peroxidation and NO generation, resulting in better cognitive impairment and antioxidant status in Aβ_25–35_-induced rats [101]. Of note, curcumin and resveratrol treatment showed decreased oxidative stress by inhibiting ROS generation, suppressed hyperphosphorylation of tau protein at threonine (T) 181 and T205, and protected SH-SY5Y cells from AβO damage [102].

**Table 1:** In vitro and in vivo effects of several plant extracts in neurodegenerative disorders.

Plant	Plant Part/Extract	Type of Study	Effects	Ref.
*Viccinium* genus (blueberries)	Fruits and leaves (methanol/water/formic acid 60:37:3 *v*/*v*/*v*)	In vivo (C57BL/6 mice)	Protected microglia cells, curtail the signs of neuroinflammation.	[19]
*Acacia dealbata*	Flower Ethanol/water	In vitro	Antioxidant Anti-AChE	[20]
*Sophora secundiflora* and *Sophora tomentosa*	Leaves Ethyl acetate and methanol	In vivo (Rats)	Antioxidant Anti-AChE	[21]
*Piper divaricatum*	Leaves Essential oil	In vitro	Anti-AChE	[22]
*Lavandula stoechas*	Aerial parts Methanol	In vivo (Swiss albino mice)	Antioxidant Anti-AChE	[23]
*Elatostema papillosum*	Leaves Methanol	In vivo (Wistar albino rats)	Anti-AChE Anti-BChE Antioxidant	[24]
*Evolvulus alsinoides*	Leaves Methanol and water	In vitro (SH-SY5Y cell-line)	Antioxidant Anti-AChE	[25]
*Psychotria calocarpa*	Leaves Methanol	In vitro and in vivo (Swiss albino mice)	Antioxidant	[26]
*Morus alba*	Leaves Water	In vivo (Swiss albino mice)	Anti-AChE, Anti-BChE Antioxidant	[27]
*Bauhinia coccinea*	Stems Ethanol	In vitro (HT22 neuronal cell line)	Anti-AChE Anti-BChE Antioxidant anti-amyloid-β (Aβ)	[28]
*Enhydra fluctuans*	Stems and leaves Chloroform	In vivo (Swiss albino mice)	Anti-AChE, Anti-BChE Antioxidant	[29]
*Dillenia suffruticosa*	Leaves Methanol	In vitro (Caenorhabditis elegans)	Anti-AChE Anti-BChE Antioxidant	[30]
*Rosmarinus officinalis*	Whole plant Ethanol, ethyl acetate and water	In vitro	Anti-AChE Antioxidant	[31]
*Origanum vulgare*	Aerial parts Ethanol	In vitro	Antioxidant Anti-apoptotic	[33]
*Bacopa floribunda*	Leaves Ethanol/water	In vivo (BALB/c mice)	Suppression of oxidative stress, neuroinflammation, and microgliosis	[34]
*Cyperus rotundus* and Zingiber officinale	Aerial parts Ethanol/methanol	In vivo (Wistar rats)	Reduced oxidative stress and AChE levels	[36]
*Typha domingensis*	Whole dried plant parts Methanol and hexane	In vitro	Anti-AChE Anti-BChE Antioxidant	[37]
*Annona cherimola*	Fruits Methanol	In vitro	Anti-AChE	[38]
*Syzygium antisepticum*	Leaves Ethanol/methanol	In vitro	Anti-AChE Antioxidant	[39]
*Dracaena reflexa*	Aerial parts and roots Methanol, butanol, and hexane	In vitro	Anti-tyrosinase Anti-AChE, Antioxidant	[40]
*Solanum macrocarpon* (L.)	Leaves Methanol and ethyl acetate	In vitro	Anti-AChE Antioxidant	[42]
*Bruguiera gymnorhiza* (L.)	Leaves and roots Water	In vitro	Anti-tyrosinase Anti-AChE Anti-BChE Antioxidant	[44]
*Artemisia scoparia*, *Artemisia*	Leaves Water	In vitro	Anti-BChE	[45]
*Mentha pulegium* (L.)	Whole plant parts Water and methanol	In vitro	Anti-AChE, Anti-BChE Antioxidant	[46]
*Lawsonia inermis*	Fruits Methanol and ethyl acetate	In vitro	Anti-BChE Antioxidant	[47]
*Ferula ammoniacum*	Aerial parts Ethanol and methanol	In vivo (Swiss albino mice)	Anti-AChE Anti-BChE Antioxidant	[48]
*Ginkgo biloba*	Fruits Ethanol, butanol, and dichloromethane	In vitro	Antioxidant	[49]
*Salvia eriophora*	Leaves Methanol and water	In vitro	Anti-AChE Anti-BChE Antioxidant	[51]
*Origanum majorana*, *Origanum onites*, *Origanum syriacum*, *Origanum hirtum*	Whole plant Ethanol	In vitro	Anti-AChE Anti-BChE	[52]
*Folium perseae*	Leaves Ethanol	In vitro	Antioxidant	[53]
*Petroselinum crispum*	Leaves Water	In vivo (Wistar albino rats)	Anti-AChE Antioxidant Anti-apoptotic	[58]
*Guazuma ulmifolia*, *Limonium brasiliense*, *Paullinia cupana*, *Poincianella pluviosa*, *Stryphnodendron adstringens* and *Trichilia catigua*	Crude plant extract and ethyl acetate extract	In vitro (SH-SY5Y cell-line)	Anti-AChE, Antioxidant	[60]
*Ocimum basilicum* (L.)	Leaves Methanol and water	In vivo (mice)	Anti-AChE Antioxidant Anti-apoptotic	[61]
*Stenocereus pruinosus*	Aerial parts Methanol	In vitro	Anti-amyloid	[95]
*Pandanus amaryllifolius*	Leaves Crude alcoholic extract	In vitro (SH-SY5Y cell-line)	Anti-amyloid β	[98]
*Carthamus tinctorius* (L.) and *Taraxacum coreanum*	Dry seeds Water	In vivo (mice)	Inhibit β-secretase and γ-secretase activity in Aβ_25–35_-infused mice.	[99]
*Cirsium japonicum*	Aerial parts Ethanol	In vivo (mice)	Antioxidant activity and attenuated lipid peroxidation and NO production.	[101]

**Table 2:** In vitro and in vivo effects of various phytochemicals against neurodegenerative disorders.

Phytocompound	Structure	Plant	Study	Molecular Mechanism	Ref.
β-caryophyllene	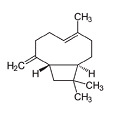	*Cannabis sativa*	In vivo (Swiss albino mice)	Neuroprotection by abrogating apoptosis through increased expression of bcl-2 and TrkB and suppression of bax and caspase-3	[55]
Sinensetin	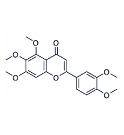	*Citrus sinensis*	In vitro (SH-SY5Y cell-line)	In vivo and in vitro anti-inflammatory, antioxidant, and antiapoptotic activities against amyloid beta (Aβ_25–35_)-induced neurotoxicity	[56]
Stigmasterol	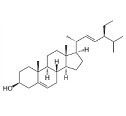	*Calotropis gigantean*	In vitro (SH-SY5Y cell-line)	Hampered apoptosis induction by suppressing ROS production and upregulated bcl-2 and FoX3a and catalase	[57]
Rosmarinic acid and ursolic acid	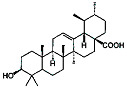	*Clinopodium revolutum*	In vivo (BALB/c mice)	Improves spatial and recognition memory	[63]
Gastrodin	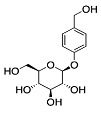	*Gastrodia elata*	In vivo (rats)	Prevented apoptosis induction by down-regulation of bax and alleviated autophagy by inhibiting beclin-1 and LC3-II	[64]
Quercetin	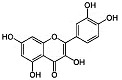	*Citrus* plants	In vivo (mice)	Inhibited cell death and degeneration by down-regulation of IL-6 and TNF-α	[66]
Asiaticoside	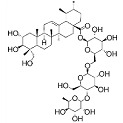	*Centella asiatica*	In vivo (Sprague-Dawley rats)	Elevated beclin-1 expression and decreased mTOR phosphorylation	[68]
Rosiridin	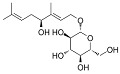	*Rhodiola rosea*	In vivo (Wistar rats)	Anti-inflammatory, antioxidant, and anti-apoptotic	[69]
Rehmannioside A	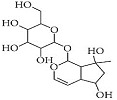	*Glutinous rehmannia*	In vivo (Sprague-Dawley rats)	Anti-inflammatory, antioxidant, and anti-apoptotic	[71]
Tilianin	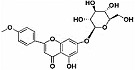	*Dracocephalum moldavica*	In vivo (Sprague-Dawley rats)	Anti-neurodegenerative, antioxidant, and anti-apoptotic	[73]
Kaempferol	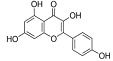	*Camellia sinensis*	In vivo (Wistar rats)	Anti-inflammatory, antioxidant, and anti-apoptotic	[74]
Marinoid J	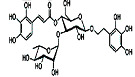	*Morinda lucida*	In vivo (Sprague-Dawley rats)	Reducing MDA level and NO activity	[75]
Morin	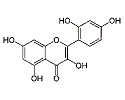	*Moraceae family*	In vivo (Sprague-Dawley rats)	Neuroprotection by antioxidation, anti-aggregation, and anti-inflammatory mechanism	[76]
Glycyrrhizic acid	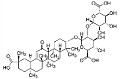	*Glycyrrhiza glabra*	In vivo (Sprague-Dawley rats)	Antioxidant activity by inhibition of ROS production and cyt-c activity	[79]
Tyrosol and hydroxytyrosol	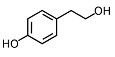	*Olea europaea*	In vivo (APP/PS1 mice)	Anti-AβO aggregation and inhibition of caspase-3 activation	[80]
Hydroxytyrosol	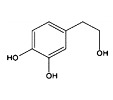	*Olea europaea*	In vivo (APP/PS1 mice)	Reverse the deregulation of JAK2/STAT3, PI3K/Akt, ERK-MAPK, and JNK-p38 signalings	[81]
Honokiol	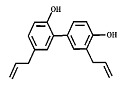	*Magnolia officinalis*	In vivo (mice)	Suppress apoptosis and neuronal damage in CA1 region of hippocampus and inhibit ROS production through attenuation of NF-κB signaling pathway	[82]
Ferrulic acid	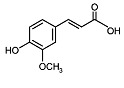	Commelinid plants	In vivo (mice)	Anti-AβO, diminished cognitive impairment and exerted antioxidant effects by activating Nrf2	[83,84]
Ferrulic acid	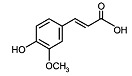	-	In vivo (Sprague-Dawley rats)	Antioxidant and neuroprotection against Aβ_1–42_-induced neurotoxicity	[85,86]
Gastrodin	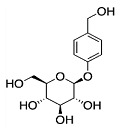	-	In vivo (Sprague-Dawley rats)	Suppresses deposition of Aβ_1–40_ and Aβ_1–42_ plaques in plasma and hippocampus of 2-VO rats by inhibiting phosphorylation of tau and amyloid β	[87]
Naringenin	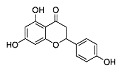	*Citrus* plants	In vitro (Neuro2a cells) In vivo (mice)	Neuroprotection against Aβ_1–42_ evoked neurotoxicity by restoring AMPK level	[88]
Pocahemiketone A	-	*Pogostemon cablin*	In vitro (SH-SY5Y cell line)	Targets NLRP3-dependent pyroptosis and oxidative stress	[89]
Myricetin	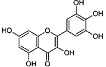	*Vitis vivifera*	In vitro (SH-SY5Y cell line) and in vivo (mice)	Anti-apoptotic and anti-AChE	[90]
Sulforaphane	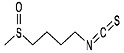	*Cruciferae* family	In vivo (C57BL/6 mice)	Neuroprotection	[91]
Procyanidin B3	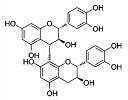	*Elaeagnus glabra f. oxyphylla*	In vitro	Anti-Aβ aggregation effects	[92]
Procyanidin B4	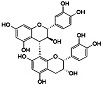	*Elaeagnus glabra f. oxyphylla*	In vitro	Anti-Aβ aggregation effects	[92]
Helichrysoside	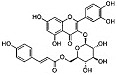	*Elaeagnus glabra f. oxyphylla*	In vitro	Anti-Aβ aggregation effects	[92]
Rosmarinic acid	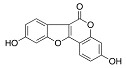	*Salvia fruticosa*	In vitro (SH-SY5Y cell-line)	Neuroprotection against Aβ_1–42_ induced neurotoxicity by down-regulating GSK3β and β-secretase	[93]
Coumestrol	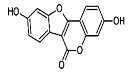	*Glycine max*	In vitro	Anti-Aβ aggregation and selective inhibition of monoamine oxidase activation	[96]
Ginsenoside F1	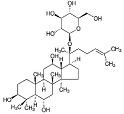	*Aralia nudicaulis*	In vivo (APPswe/PSEN1dE9 double-transgenic mice) and In vitro (Neuro2a and SH-SY5Y cell lines)	Anti-Aβ aggregation	[97]
β-caryophyllene	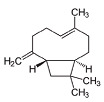	-	In vitro (NSC-34 cells)	Antioxidant and anti-apoptotic activities by inhibiting Aβ aggregation	[100]
α-bisabolol	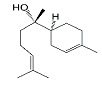	*Gochnatia polymorpha*	In vitro (NSC-34 cells)	Antioxidant and anti-apoptotic activities by inhibiting Aβ aggregation	[100]
Curcumin	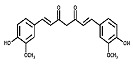	*Curcuma longa*	In vitro (SH-SY5Y cell-line)	Antioxidant activity by inhibited ROS generation	[102]
Resveratrol	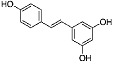	*Vaccinium species*	In vitro (SH-SY5Y cell-line)	Antioxidant activity by inhibited ROS generation	[102]

## 4. Plant-Derived Bioactive Compounds and Combinatorial Approaches for the Management of Neurodegenerative Disorders

In recent years, combination regimens have been explored in which researchers use plant-derived bioactive compounds in conjunction with other drugs to achieve higher efficacy and overcome the toxicities associated with the standard drugs of neurodegenerative diseases. Currently, drugs such as donepezil, amantadine, galantamine, apomorphine, rasagiline, riluzole, baclofen, entacapone, memantine, pramipexole, rivastigmine, carbidopa, dantrolene, and ropinirole are prescribed to treat different neurodegenerative disorders and associated problems. However, most of these drugs cause mild to severe side effects. In this section of the review, we included studies on donepezil, galantamine, and huperzine A combined with other phytocompounds.

Donepezil is prescribed by clinicians for treating dementias, but the medicine does not cure dementia. However, the drug may provide symptomatic treatment for AD, PD, and Lewy body dementia. A previous in vivo study showed that consumption of extra-virgin olive oil rich in oleocanthal enhanced the effects of donepezil through significant reduction of amyloid β aggregation by regulating synaptic proteins and reducing neuroinflammation [103]. Donepezil and Ginkgo ketoester tablet combination exerts antiamnesic effects via antioxidation and concentration-dependent inhibition of acetylcholinesterase and butyrylcholinesterase, which have been closely linked to both the inactivation of brain-derived neurotrophic factor (BDNF) and tyrosine protein kinase B (TrkB) signaling pathways, as well as effects on oxidative stress and cholinergic pathway [104]. In a human study, 96 outpatients of AD were enrolled and each of them was administered *Ginkgo biloba* extract (EGb) (240 mg/day) and donepezil (5 mg/day for four weeks and then 10 mg/day) for 22 weeks, and the results suggested that combination therapy is superior to donepezil monotherapy and has lower side effects [105]. An in vitro study showed that caffeic acid (0.075 mg/mL) combined with donepezil (0.025 mg/mL) inhibited acetylcholinesterase and butyrylcholinesterase significantly, and donepezil (0.050 mg/mL) and caffeic acid (0.050 mg/mL) combination inhibited sodium nitroprusside-induced lipid peroxidation in rat brain homogenate the most [106]. Likewise, the activity of superoxide dismutase (SOD) and the content of malondialdehyde (MDA) were measured in resveratrol and donepezil-treated Wistar rats (colchicine-induced AD). The findings of the in vivo study revealed an improved antioxidant status, which was due to enhanced SOD activity in animal groups treated with donepezil and resveratrol combinations, implying that both drugs work synergistically [107]. In another in vivo study, Wistar rats were administered with donepezil alone and in combinations of donepezil and gallic acid, and the findings show that the combination of donepezil (10 mg/kg) and gallic acid (50 mg/kg) rectified rising MDA levels and brain activities of AChE and BChE, and the combination improved activities of SOD and catalase with a concomitant increase in thiol level, indicating synergistic effects of donepezil and gallic acid against AlCl_3_-induced neurotoxicity [108]. 

An interesting study used SIP3, a mixture of *Santalum album*, *Illicium verum*, and *Polygala tenuifolia*, and donepezil combinations, and the results showed improved memory and depression in a mouse model of AD as well as in vitro [109]. Pretreatment with quercetin significantly potentiated the efficacy of donepezil, evidenced through the improved cognitive memory in scopolamine-induced amnesia rats, which could have been due to the reduced levels of AChE and Aβ1–42 as well as elevated glutathione level, decreased lipid peroxidation, and reversal of neuronal damage in the treated groups [110]. Earlier, *Stephaniae tetrandrae* radix (STR), a commonly used traditional Chinese medicine, was widely used in the treatment of rheumatism, edema, and dysuria. Recently, STR was investigated to identify the pharmacologically important small compounds as well as their role in the neurodegenerative disorders, including AD. The study revealed that STR contains three alkaloids, cyclanoline, fangchinoline, and tetrandrine, as its major phytoconstituents. Among the three alkaloids, cyclanoline and fangchinoline suppressed AChE, but tetrandrine had no such enzymatic inhibition. However, pharmacological combinations of fangchinoline and huperzine A or donepezil inhibited AChE more than their individual treatments, implying synergistic effects [111]. Caffeine, an alkaloid, is the world’s most consumed dietary component and is also regarded as a strong central-nervous-system stimulant. It is found mostly in coffee beans and *Camellia sinensis* leaves. In a recent study, caffeine’s effects, either alone or in conjunction with donepezil, were investigated. Caffeine potentiated donepezil’s actions in vitro by enhancing AChE inhibition and antioxidation. Co-administration of caffeine (50 or 100 mg/kg) and donepezil (5 mg/kg) inhibited AChE more effectively than in single dosages, although low caffeine consumption potentiated donepezil’s antioxidant properties but had little effect on its inhibitory activity against AChE [112]. Harmine, an alkaloid derived from *Peganum harmala*, has been studied in vitro and in vivo for many pharmacological activities. Harmine has the capability to cross the blood–brain barrier and inhibit AChE activity [113]. Harmine (20 mg/kg) and donepezil (3 mg/kg) co-administration resulted in significant shortened escape latency and path length and improved memory in scopolamine-pretreated transgenic mice, which was linked to AChE inhibition in the cerebral cortex of mice. However, it was observed that the combination of harmine and donepezil did not inhibit Aβ protein aggregation in the hippocampus of scopolamine-treated and transgenic mice [114].

Genistein is a plant-derived isoflavone profoundly present in *Glycine max*, which has been investigated for its role in different diseases, including cancer and neurodegenerative diseases. In a recent study, genistein and galantamine combination was assessed in order to explore its neuroprotective effects against Aβ1–42-induced toxicity in AD, which was due to decreased genotoxicity and cell death [115]. In comparison to placebo and other cholinesterase inhibitors such as galantamine and donepezil, EGb7 (*Gingko biloba* extract) and memantine (cholinesterase inhibitor) were shown to be ineffective [116]. *Huperzia serrata* is an interesting medicinal plant used in traditional medication in Asia for treating cognitive impairment, dementia, and schizophrenia, and one of its compounds, huperzine A, is a well-established competitive reversible inhibitor of AChE. Huperzine A is a sesquiterpene alkaloid derived from *Huperzia serrata* that has been studied for its psychopharmacological effects, especially cognitive performance and neuroprotective effects that may be useful in the treatment of neurodegenerative disorders. Previously, it was reported that caffeic acid and ferrulic acid have the ability to potentiate huperzine A-mediated neuroprotective effects. Furthermore, they suggested that huperzine A in combination with either caffeic acid or ferrulic acid did not potentiate AChE inhibition in AD and memory deficits [117]. In a preclinical trial, memantine and huperzine A combination was identified as a superior AChE inhibitor than three other AChE inhibitors, including donepezil, rivastigmine, and galantamine. All four AChE inhibitors were effective in improving the mini-mental-state-examination (MMSE) and activities-of-daily-living (ADL) scores of AD patients when used in conjunction with memantine. However, huperzine A demonstrated superior effectiveness only when compared to other AChE inhibitors [118]. The antioxidant EGCG from *Camellia sinensis* (green tea) was investigated to see whether it could enhance the inhibitory effects of huperzine A on AChE activity in Alzheimer’s disease. Notably, the study’s findings revealed that EGCG is a poor inhibitor of AChE within a range of 10 to 300 mg/kg; however, EGCG addition substantially enhanced the inhibitory effects of huperzine A on AChE activity in AD, which might be attributed to EGCG’s antioxidant action [119]. Other studies demonstrated that the combination of huperzine A with *Convolvulus pluricaulis* and *Celastrus paniculatus* improves cognitive function and health. The combinations of huperzine A with *Convolvulus pluricaulis* and *Celastrus paniculatus* showed better efficacy and synergistic AChE inhibition. However, no significant adverse toxic events were reported upon administering drug combinations [120].

## 5. Plants and Phytochemicals under Clinical Trials

Neurodegenerative disorders are debilitating and disabling, and their prevalence is increasing as the population ages. Therefore, the discovery of any therapeutics that stop or slow disease progression will help lessen the psychological and socio-economic burden. Notably, oxidative stress and inflammation are well-studied phenomena. Oxidative stress arises as a consequence of an imbalance between reactive oxygen species generation and the antioxidant system. A plethora of evidence clearly indicates that oxidative stress and mitochondrial dysfunction play a significant role in disease pathogenesis. Hence, various plant-derived compounds and their extracts targeting oxidative stress, neuroinflammation, and mitochondrial dysfunction have been documented to hold great promise in preclinical studies. In multitudinous preclinical studies, many plant extracts and their bioactive compounds have been demonstrated to be efficacious in treating neurodegenerative disorders, including AD, PD, TBI, and other diseases. These plant extracts and plant-derived bioactive compounds have great antioxidant, anti-apoptotic, and anti-inflammatory potential, which may help in neuroprotection. However, there is a significant gap in the clinical translation of these plant extracts and plant-derived bioactive compounds. For instance, according to a double-blinded phase II clinical trial (NCT01504854), resveratrol from red wine and the skin of red grapes may lower the risk of dementia by activating sirtuin proteins in aging and AD. Ethnodyne is a food supplement that is composed of plant-derived active components. In a clinical trial (NCT02815800), ethnodyne and vitamin B2 were tested against PD, and the results suggested that the combination improved motor and non-motor signs of the disease. Guar beans, rich in gum and fiber, have previously been shown to be effective in limiting inflammation and delaying the onset and progression of multiple sclerosis [121]. Chronic constipation commonly affects the gastrointestinal health of PD patients, and in a recent clinical trial (NCT04569656), guar gum rich in galactomannan from the seeds of *Cyamopsis* Tetra-Gonolobus prevented bloating, flatulence, and meteorism. Cannabidiol from *Cannabis sativa* has been investigated for numerous pharmacological properties, including neuroprotective and neuromodulatory effects, and serves as an alternative approach to treating different CNS disorders [122]. In a randomized, double-blinded, placebo-controlled phase II clinical trial (NCT02818777), the tolerability and efficacy of cannabidiol were studied on tremors in PD.

Sulforaphane from cruciferous plants has been presented as an epigenetic modulator, antioxidant, and anti-inflammatory agent, and its usefulness in dopaminergic neuronal survival is well established. In a randomized phase II clinical trial (NCT05084365), sulforaphane was supplied to 100 PD patients for 24 weeks and was assessed for its safety, tolerability, cognitive functions, and motor symptoms. Acetylcholine is the key regulatory molecule for the transmission of messages between nerve cells, and it is being degraded by acetylcholinesterase. ZT-1 is a plant-derived extract that is used in China for the treatment of memory disorders. It mainly acts by blocking the activity of acetylcholinesterase, and thus ZT-1 restores acetylcholine levels. In a randomized, double-blinded clinical trial (NCT00423228), ZT-1 along with donepezil was investigated for its safety and efficacy in AD. SCI-110 is a drug combination of dronabinol (a synthetic analogue of tetrahydrocannabinol from the *Cannabis* plant) and palmitoylethanolamide (PEA). In a phase II clinical trial (NCT05239390), combinations of dronabinol (2.5–12.5 mg) and 800 mg PEA were supplied to AD patients and studied for their tolerability, safety, appetite, and sleep quality. NIC5-15 is a naturally occurring small compound found in soy and other plants, and it was shown in a randomized, double-blind, phase II clinical trial (NCT01928420) to inhibit β-amyloid accumulation in AD. In a clinical trial (NCT04812418), combination extracts of the plants eschscholtzia (120 mg) and valerian (50 mg) were supplied in the form of tablets to study their efficacy against anxiety and sleep disorders. The effects of dietary strawberry supplementation on age-related problems were studied in a clinical trial (NCT02051140), and the results showed that it improved cognition and mobility by protecting against oxidative stress and inflammation. Similarly, in another clinical trial (NCT01888848), dietary blueberry supplementation improved cognition and mobility by protecting against oxidative stress and inflammation. In a clinical trial (NCT05609890), the safety and efficacy of many plant formulations (saffron, lemon balm, valerian, and tea extract) were assessed for sleep quality. This indicates the medicinal importance of plant extracts against neurodegenerative disorders, but there are very few studies on plant-derived compounds with regards to neurodegenerative disorders.

## 6. Conclusions, Challenges, and Future Directions

Current available drugs have been documented to exert several severe side effects, and disease complexity also worsens the patient’s health even after treating the patient with standard symptomatic treatments, which limits the scope of the standard therapies against neurodegenerative disorders. Despite the promising results reported by several researchers, there is still a knowledge gap in terms of pharmacokinetics and pharmacodynamics of several plant-derived bioactive compounds in human clinical studies. In addition, critical clinical-translation challenges include dose optimization, product consistency, and quality control of the plant formulations and plant-derived bioactive compounds for different neurodegenerative disorders. Importantly, plant formulations and plant-derived compounds benefit and, simultaneously, suffer from low bioavailability and rapid renal clearance. Moreover, plasma concentrations of such plant-derived bioactive compounds present in the diet are significantly lower than the pharmacological doses needed in vitro and in vivo to trigger neuroprotective responses. Therefore, it is reasonable to predict the pharmacological and chemopreventive doses, which may help fill the knowledge gaps in the disease severity and complexity. In past few decades, numerous preclinical studies showed that plant formulations and plant-derived compounds possess enormous chemopreventive and therapeutic potential against many neurodegenerative disorders. Therefore, there has been increasing interest in identifying chemopreventive and therapeutic formulations and bioactive compounds obtained from plants that could lead to the development of novel therapeutic options to curb the increasing burden of neurodegenerative disorders throughout the world. Notably, managing neurodegenerative disorders through selective plant formulations and plant-derived bioactive compounds that are capable of crossing the blood–brain barrier represents a novel, safe, affordable, and alternative option to standard therapies for the prevention and treatment of neurodegenerative disorders. Therefore, this review integrates recent in vitro and in vivo experimental data that suggest the potential plant formulations and plant-derived bioactive compounds targeting molecular culprits involved in abnormal protein dynamics, neuroinflammation, and oxidative stress in neurodegenerative diseases. Due to their anti-cholinesterase, antioxidant, anti-inflammatory, anti-aggregation, and anti-apoptotic effects, plant formulations as well as plant-derived bioactive compounds may provide huge chemopreventive and therapeutic benefits in the drug development against neuroinflammation, oxidative stress, protein aggregation, and apoptosis induction in neuronal cells. Herein, we also review in vitro and in vivo bioactivities and critically appraise the most plausible active plant-derived bioactive compounds.In addition, we summarize cellular and molecular targets and mechanisms of action of plant-derived bioactive compounds. Investigations on plant formulations and plant-derived bioactive compounds revealed that plant-based diets are a rich source of flavonoids, terpenes, phenolics, carotenoids, sterols, and anthocyanins as well as vitamins and minerals. Hence, consuming a plant-based diet that contains such phytochemicals may lead to potential health benefits, including neuroprotection, hepatoprotection, cardioprotection, and others.

## Data Availability

All the Data are Available in the manuscript.
